# Cost-effectiveness of the implementation of [^68^Ga]Ga-PSMA-11 PET/CT at initial prostate cancer staging

**DOI:** 10.1186/s13244-022-01265-w

**Published:** 2022-08-13

**Authors:** Esmée C. A. van der Sar, Willem R. Keusters, Ludwike W. M. van Kalmthout, Arthur J. A. T. Braat, Bart de Keizer, Geert W. J. Frederix, Anko Kooistra, Jules Lavalaye, Marnix G. E. H. Lam, Harm H. E. van Melick

**Affiliations:** 1 Department of Radiology and Nuclear Medicine, University Medical Center Utrecht, Heidelberglaan 100, 3584 CX Utrecht, The Netherlands; 2 Julius Center, University Medical Center Utrecht, Utrecht, The Netherlands; 3 Department of Radiotherapy, University Medical Center Utrecht, Utrecht, The Netherlands; 4 Department of Urology, Meander Medical Center, Amersfoort, The Netherlands; 5 Department of Nuclear Medicine, St Antonius Hospital, Nieuwegein, The Netherlands; 6 Department of Urology, St Antonius Hospital, Nieuwegein, The Netherlands

**Keywords:** Prostate cancer, PSMA PET/CT, Cost-effectiveness, Radioligand, Gallium

## Abstract

**Background:**

Despite its high specificity, PSMA PET/CT has a moderate to low sensitivity of 40–50% for pelvic lymph node detection, implicating that a negative PSMA PET/CT cannot rule out lymph node metastases. This study investigates a strategy of implementing PSMA PET/CT for initial prostate cancer staging and treatment planning compared to conventional diagnostics. In this PSMA PET/CT strategy, a bilateral extended pelvic lymph node dissection (ePLND) is only performed in case of a negative PSMA PET/CT; in case of a positive scan treatment planning is solely based on PSMA PET/CT results.

**Method:**

A decision table and lifetime state transition model were created. Quality-adjusted life years and health care costs were modelled over lifetime.

**Results:**

The PSMA PET/CT strategy of treatment planning based on initial staging with [^68^Ga]Ga-PSMA-11 PET/CT results in cost-savings of €674 and a small loss in quality of life (QoL), 0.011 QALY per patient. The positive effect of [^68^Ga]Ga-PSMA-11 PET/CT was caused by abandoning both an ePLND and unnecessary treatment in iM1 patients, saving costs and resulting in higher QoL. The negative effect was caused by lower QoL and high costs in the false palliative state, due to pN1_lim_ patients (≤ 4 pelvic lymph node metastases) being falsely diagnosed as iN1_ext_ (> 4 pelvic lymph node metastases). These patients received subsequently palliative treatment instead of potentially curative therapy.

**Conclusion:**

Initial staging and treatment planning based on [^68^Ga]Ga-PSMA-11 PET/CT saves cost but results in small QALY loss due to the rate of false positive findings.

**Supplementary Information:**

The online version contains supplementary material available at 10.1186/s13244-022-01265-w.

## Key points

- A negative [^68^Ga]Ga-PSMA-11 PET/CT cannot rule out lymph node metastases.
- A positive [^68^Ga]Ga-PSMA-11 PET/CT may replace an extended pelvic lymph node dissection .
- Initial prostate cancer staging with [^68^Ga]Ga-PSMA-11 PET/CT saves health care cost.
- [^68^Ga]Ga-PSMA-11 PET/CT may result in minor loss in quality of life.
- Loss in quality of life due to false positive findings [^68^Ga]Ga-PSMA-11 PET/CT.

## Background

Adequate staging of intermediate- to high-risk prostate cancer is of great importance for definite treatment planning and prognosis. To detect metastases, conventional imaging (X-ray computed tomography (CT), magnetic resonance imaging (MRI) and skeletal scintigraphy) and a bilateral extended pelvic lymph node dissection (ePLND) are the traditional diagnostic work-up [1].

However, an ePLND is an invasive, costly and potentially harmful procedure with complications including lymphocele 0.1–10.6% [2, 3], thrombosis 0–8% [2, 3] and nerve injury 0–1.8% [3] and commonly requiring overnight hospital admission [2]. The use of ePLND is primarily diagnostic [1, 4], for which a reliable non-invasive cost-effective alternative for metastatic prostate cancer is desirable.

In recent years, “prostate specific membrane antigen” (PSMA) PET/CT has rapidly evolved in prostate cancer imaging. Compared to conventional imaging, PSMA PET/CT has a higher specificity of approximately 90% in the detection of pelvic lymph node metastases in men with newly diagnosed prostate cancer [5–7]. There is also a 27% greater accuracy in distant metastases detection (sensitivity 85% and specificity 98%) [5]. PSMA PET/CT has also shown to be less costly than conventional imaging; therefore, it can be expected that PSMA PET/CT would be cost-effective in comparison with conventional imaging [8].

However, despite its high specificity, PSMA PET/CT has a moderate to low sensitivity of 40–50% for pelvic lymph node detection [5–7], implicating that a negative PSMA PET/CT cannot rule out lymph node metastases and that for adequate prostate cancer staging an ePLND is still needed.

Earlier cost-effectiveness research showed that using PSMA PET/CT instead of ePLND for pelvic lymph node detection was likely to save costs but reduced quality of life (QoL). This was mainly because of false positive findings by PSMA PET/CT [9, 10]. However, these analyses did not incorporate the positive effect of distant metastases detection and did not include the high false negative rate (low sensitivity) for pelvic lymph node detection.

This study aims to investigate a strategy of implementing PSMA PET/CT for initial prostate cancer staging and treatment planning instead of conventional diagnostic work-up (i.e. standard ePLND). In this investigated PSMA PET/CT strategy, ePLND is only performed in case of a negative PSMA PET/CT (due to the low sensitivity); in case of a positive scan treatment planning is solely based on PSMA PET/CT results.

## Methods

### Patient cohort

**Fig. 1:**
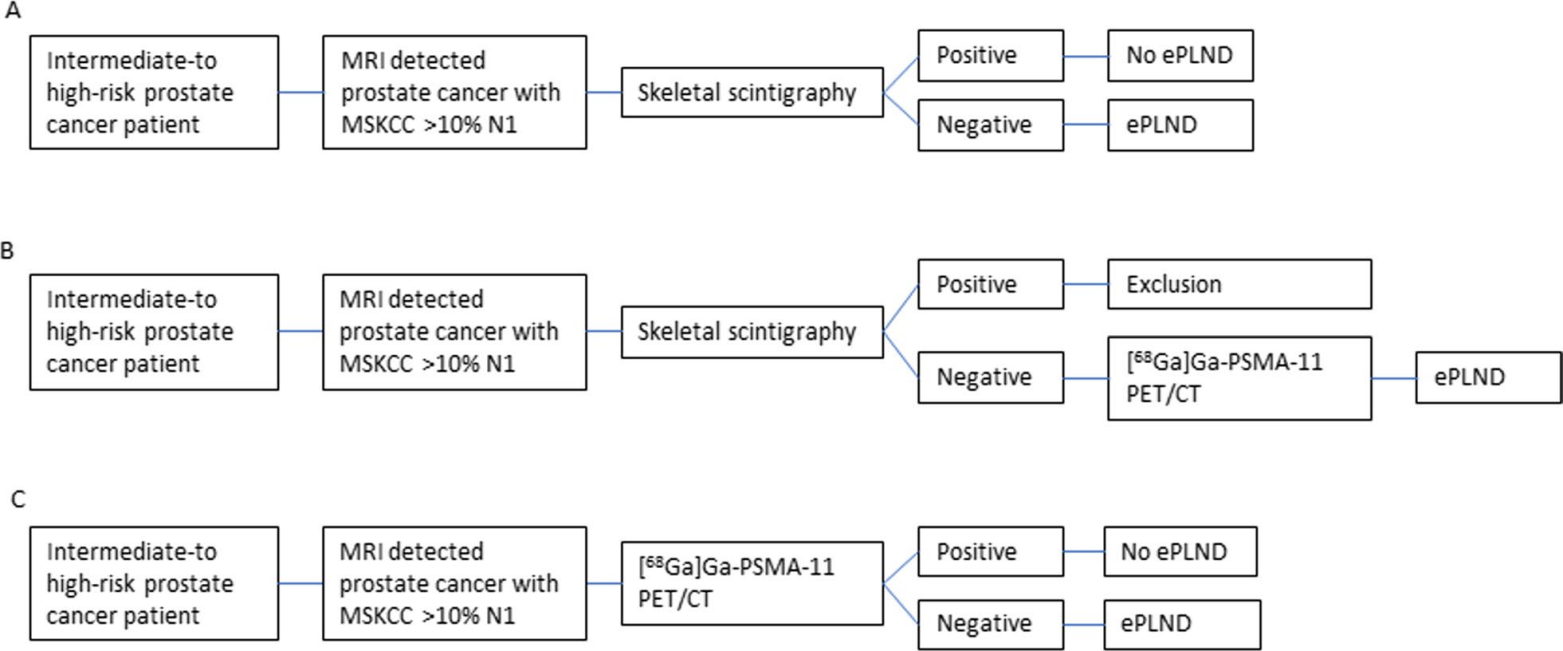
Flow chart of initial prostate cancer staging and treatment planning in standard of care, PEPPER-study and in the potential PSMA PET/CT strategy. **A**: Standard of care. **B**: PEPPER-study. **C**: PSMA PET/CT strategy (skeletal scintigraphy was replaced by [^68^Ga]Ga-PSMA-11 PET/CT and no ePLND in case of positive iN1 and iM1 findings on [^68^Ga]Ga-PSMA-11 PET but only ePLND in case of negative PSMA). *ePLND* Extended pelvic lymph node dissection, MRI magnetic resonance imaging, *MSKCC* Memorial Sloan Kettering Cancer Center, *PET/CT* positron emission tomography/computed tomography, *PSMA* prostate specific membrane antigen

Data from the PEPPER-study (NTR6830) was used, which evaluated the diagnostic performance of [^68^Ga]Ga-PSMA-11 PET/CT for initial prostate cancer staging in a prospective study. Patients with a positive skeletal scintigraphy were excluded (Fig. 1).

For our cost-effectiveness analysis, patients were categorized as: no lymph node metastasis (N0), limited lymph nodes metastasis defined as ≤ 4 pelvic lymph node metastases (N1_lim_), extended lymph nodes metastasis defined as > 4 pelvic lymph node metastases (N1_ext_), distant metastasis (M1) defined as extra pelvic lymph node metastasis (M1a), or bone and/or visceral metastasis (M1b/c). Ground truth for N-status was always based on pathology results, but for distant metastases this was based on a combination of pathology, additional imaging and/or follow-up (Additional file 1: Fig. S1) [7].

### Costs, utilities, disutilities and yearly probabilities

Yearly probabilities, costs and disutilities of treatment procedures were derived from the literature or from internal sources (Additional file 1: Table S1). QoL (utility) values were expressed as quality-adjusted life years (QALY). A QALY of one indicates one year in best possible health, a QALY of zero indicates death. Disutilities express QALY decrement. Costs were expressed as 2020 price levels using the Dutch national price index [11]. No exact data on the impact of ePLND on QoL was found. Using literature and expert opinion, the disutility of this procedure was estimated (Additional file 1: Table S1).

### Model development

#### Decision table

Short-term costs and QoL of diagnosis and treatment of prostate cancer patients were calculated using a decision table (Table 1). This included all possible scenarios for both PSMA PET/CT strategy and standard of care. Diagnostic accuracy was calculated using the frequency outcomes from the PEPPER-study (Additional file 1: Fig. S1). Subsequently, the treatment scheme was obtained using the standard of treatment given the diagnostic outcomes (Table 1). After treatment, the patients transit towards one of four health stages: (NEOD (coming from N0 or N1)), palliative and false palliative (pN1_lim_ patients being falsely diagnosed as iN1_ext_).

**Table 1:** Decision table based on the diagnostic outcomes of the PEPPER-study cohort

Ground truth	Diagnosis	Patients (n)	Frequency (%)	SE (%)	ePLND spared (y/n)	Diagnostic scheme	Curative treatment scheme	Health state
[^68^Ga]Ga-PSMA-11 PET/CT scenario								
*N0 patients*								
pN0	pN0	49	91%	-	No	*GPP* + *MRI* + *ePLND*	*RT/RP*	NEOD-N0
pN0	iN1_lim_	5	9%	3.9%	Yes***	*GPP* + *MRI*	*RT/RP* + *Pelvic RT* + *ADT*	NEOD-N0
pN0	iN1_ext_	0	NA*****	-	Yes**	*NA*	*NA*	*NA*
*N1*_*Lim*_* patients*								
pN1_lim_	pN0	24	65%	7.8%	No*	*GPP* + *MRI* + *ePLND*	*RT/RP* + *Pelvic RT* + *ADT*	NEOD-N1
pN1_lim_	iN1_lim_	12	32%	-	Yes	*GPP* + *MRI*	*RT/RP* + *Pelvic RT* + *ADT*	NEOD-N1
pN1_lim_	iN1_ext_	1	2.7%	2.7%	Yes**	*GPP* + *MRI*	*NA*	False palliative
*N1*_*Ext*_* patients*								
pN1_ext_	pN0	0	NA*****	-	No*	*NA*	*NA*	*NA*
pN1_ext_	iN1_lim_	1	33%	27.2%	Yes***	*GPP* + *MRI*	*RT/RP* + *Pelvic RT* + *ADT*	Palliative
pN1_ext_	iN1_ext_	2	67%	-	Yes	*GPP* + *MRI*	*NA*	Palliative
*M1 patients*								
pM1	iM1	8	100%	-	Yes****	*GPP* + *MRI*	*NA*	Palliative
Standard of care scenario								
*N0 patients*								
pN0	pN0	54	100%	-	No	*GPP* + *MRI* + *ePLND*	*RT/RP*	NEOD-N0
N1_Lim_ Patients								
pN1_lim_	pN1_lim_	37	100%	-	No	*GPP* + *MRI* + *ePLND*	*RT/RP* + *Pelvic RT* + *ADT*	NEOD-N1
*N1*_*Ext*_* patients*								
pN1_ext_	pN1_ext_	3	100%	-	No	*GPP* + *MRI* + *ePLND*	*NA*	Palliative
M1 Patients								
pM1	pN0	8	100%	-	No	*GPP* + *MRI* + *ePLND*	*RT/RP*	Palliative

The proportion was used to define treatment costs and utilities. The patients distribution among states was used as cohort for the Markov simulation. *ADT*  Androgen deprivation therapy, *ePLND* extended pelvic lymph node dissection, GPP = [^68^Ga]Ga-PSMA-11 PET/CT, *MRI * magnetic resonance imaging, *M1*  distant metastasis including extra pelvic lymph node metastasis, bone and/or visceral metastasis, *N0*  no lymph node metastasis, *N1lim*  limited lymph nodes metastasis defined as less than or equal to four pelvic lymph node metastasis, *N1ext*  extended lymph nodes metastasis defined as more than four pelvic lymph node metastasis, *NA*  not applicable, *NEOD*  no evidence of disease, *PET/CT*  positron emission tomography/computed tomography, *PSMA*  prostate specific membrane antigen, *RP*  radical prostatectomy, *RT*  radiotherapy.

*ePLND would reveal misdiagnosis of the [^68^Ga]Ga-PSMA-11 PET/CT and therefore assuring correct treatment

**Misdiagnosis by [^68^Ga]Ga-PSMA-11 PET/CT would result in false positive palliative state and thus causing lower treatment effects

***Misdiagnosis by [^68^Ga]Ga-PSMA-11 PET/CT would result higher treatment costs for pelvic radiotherapy and ADT but equal outcomes regarding after treatment effects

****ePLND would not recognize the M1 state resulting in higher treatment costs and lower treatment utilities for these patients in the standard of care. However after treatment effects would be equal

*****It was assumed to be impossible to overestimate more than 4 lymph nodes metastases in N0 patients and vice versa

#### Lifetime state transition model

**Fig. 2:**
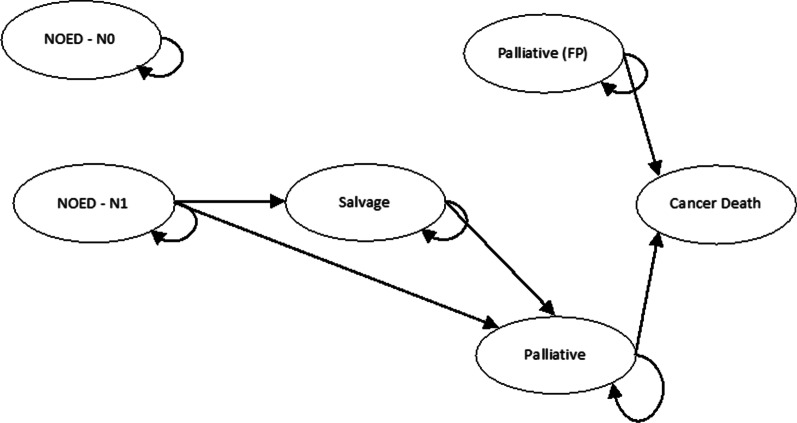
Lifetime state transition model used for the different scenarios. The model consists of six health states where patients can find themselves in during follow-up: ‘No evidence of disease after treatment of N0 disease’ (NEOD-N0), ‘No evidence of disease after treatment of N1 disease’ (NEOD-N1), ‘Salvage’, ‘Palliative’, ‘False Palliative’ and ‘Cancer death’. The NEOD states were used to reflect patients who were treated curatively. It was assumed that patients in the NEOD-N0 state would be fully cured and stay there till death. Patients in NEOD-N1 state were assumed to be at risk for biochemical recurrence (BCR), when BCR occurs they transfer towards salvage or directly towards palliative. The salvage state was designed to reflect the period of salvage initialized after BCR would occur. After salvage treatment, they either stay in salvage state or transit to palliative state. The palliative state reflects the long-term palliative period for prostate cancer patients. In this period, no curative treatments are initialized. The false palliative state was designed to mimic the palliative state of patients who are falsely being diagnosed for palliative treatment by [^68^Ga]Ga-PSMA-11 PET/CT. Patients in the palliative state and the false palliative state would stay there until death. Prostate cancer-related death could only occur in the palliative state and the false palliative state. All patients could transit to non-prostate cancer-related death from every state (these lines are hidden)

To calculate lifetime costs and QoL of treatment, a lifetime state transition model simulating patients’ follow-up was created, based on previous work of Scholte et al. [10] (Fig. 2). The health stages of the decision table are integrated in the lifetime state transition model together with two additional health states, namely salvage and (cancer) death. Yearly probabilities, cost and utility values of each transition state are shown in Table 2.

**Table 2:** Yearly input parameters of the lifetime state transition model

Parameter	Value	Distribution (SE)	Source
*Lifetime state transition model probabilities*			
Probability BCR in the NEOD-N1 (pBCR)	0.45 (Gompertz; Rate 0.66, shape -0.38)	Normal	Mandel et al. [21]
Percentage with BCR to salvage	0.63	Beta (0.063)	De Bruycker et al. [22]
N1-NEOD to salvage	pBCR * Percentage with BCR to salvage		
N1-NEOD to palliative	pBCR * (1-Percentage with BCR to salvage)		
Salvage to palliative	0.31	Beta (0.031)	Decaestecker et al. [23]
Cancer mortality (palliative)	0.032	Beta (0.0032)	Tumati et al. [24]
Cancer mortality (false palliative)	0.032	Beta (0.0032)	Assumption: equals Cancer Mortality
All-Cause mortality	Standard mortality rates age 69 and higher	Fixed	CBS [25]
*[*^*68*^*Ga]Ga-PSMA-11 PET/CT probabilities*			
pN0 to palliative (FP)	0	Fixed	PSMA PET/CT indicates multiple LNMs in N0 patients
pN1_lim_ to palliative (FP)	0.027	Beta (0.027)	PSMA PET/CT indicates multiple LNMs in N1_lim_
*Costs (€)*			
NEOD-N0 and N1	108	Gamma (€11)	De Rooij et al. [26]
(False) palliative	4,613^1^	Gamma (€1,153)	Schwenk et al. [27] FK [28]
Salvage	8,022^2^	Gamma (€802)	Schwenk et al. [27]
Palliative to death (transition cost)	16,720	Gamma (€1,672)	Tien et al. [29]
*Utilities (QALY)*			
NEOD-N0 and N1	0.81^3^	Beta (0.081)	Versteegh et al. [30]
Scholte et al. [10]			
(False) palliative	0.67	Beta (0.067)	Stewart et al. [31]; Asymptomatic spread
Salvage	0.77	Beta (0.077)	Heijnsdijk et al. [32]; RT

All-cause mortality was derived from the Dutch public data [25] regarding mortality rates for age 69 and higher in 2019. The transition parameters BCR from NOED N1, salvage to palliative and palliative to death (Cancer mortality) were determined by fitting a Gompertz or exponential distribution on the Kaplan Meier curves, using webplotdigitizer [33]. All other transitions were derived from literature. *BCR* biochemical recurrence, *CBS* centraal bureau voor statistiek (Dutch national price index), *FP* false positive, *LNM* lymph node metastases, *N0* no lymph node metastasis, *N1*_*lim*_ limited lymph nodes metastasis defined as less than or equal to four pelvic lymph node metastasis, *NOED* No evidence of disease, *pBCR* probability on biochemical recurrence, *SE* standard error, *PET/CT* positron emission tomography/computed tomography, *PSMA* prostate specific membrane antigen.

^1^Cost of palliative therapy was assumed to be the costs of 66Gr Radiotherapy and 4 shots Goserilin

^2^Cost of salvage treatment was assumed to be the mean cost of all radiotherapy options described in Schwenk et al. [27]

^3^Utility was estimated using the mean utility for men aged 70–80 and a fixed correction for long-term primary treatment complications as calculated by Scholte et al. [10]

Average age at model start was 69 years (consistent with the existing patient cohort) and the model ran until death (40 cycles; one cycle corresponded to one year). Yearly discounting percentage of 4% and 1.5% were used for costs and utility outcomes, according to Dutch guidelines [12].

Finally, total costs and QoL were calculated by adding the mean treatment cost and disutility outcomes to the lifetime model costs and QoL outcomes.

For optimal modelling, a number of assumptions were made. Firstly, during state transitioning, subjects in the NEOD-N0 state could not experience BCR. Secondly, patients in the salvage or (false) palliative state could not transit back towards NEOD. Thirdly, regarding diagnostic accuracy, it was impossible for [^68^Ga]Ga-PSMA-11 PET/CT to diagnose pN0 patients as being iN1_ext_ and vice versa. Fourthly, our model assumed that diagnosis of patients suffering from iM1a/b/c disease by [^68^Ga]Ga-PSMA-11 PET/CT was always correct.

And lastly, PSMA-M1 patients were assumed to be diagnosed as pN0 patients in standard of care, since M1 disease on conventional bone scintigraphy was an exclusion criterion in the PEPPER-study.

### Outcomes

Our main outcome: cost-effectiveness was expressed as incremental: costs, QoL (utility), life years and incremental cost-effectiveness ratios (ICERs), for [^68^Ga]Ga-PSMA-11 PET/CT versus conventional diagnostics (ePLND and skeletal scintigraphy), from a health care perspective. The ICER (€/QALY) represents the investment cost for adding one QALY. An ICER was “dominant” when the treatment increased QoL and saved costs. Conversely, an ICER was considered “dominated” when the treatment reduced QoL and increased costs. In other words, a dominant strategy is cost-effective, whereas a dominated strategy is not cost-effective. Net monetary benefit (NMB) was calculated using a willingness to pay (WTP) €80,000, according to Dutch standards [13]. The NMB translates utility values into euros, using the WTP to quantify the net worth of one incremental QALY in €. When the NMB is above zero, the intervention is more cost-effective compared to any given treatment with an ICER of €80,000/QALY, thus creating NMB.

As our main outcome is only based on a single prospective study cohort, additional cost-effectiveness analysis, using the probabilities of the Dutch population were performed. These analyses were added as Additional file 1.

Analysis was performed using R version 4.0.3 and Microsoft Excel version 16.35. An online accessible tool is available at: https://wrke.shinyapps.io/shiny_html_temp/. Technical validation was performed by peer review and by recreating the excel model in R. All inputs values were verified by experts.

### Sensitivity analysis

Three types of sensitivity analyses were performed:

Firstly, deterministic sensitivity analysis (DSA) was performed to evaluate the impact of all input parameters individually on model outcomes. All input variables were varied by ± the reported standard error (SE) value (Table 2, Additional file 1: Table S1) and cost-effectiveness result measured in NMB (WTP €80,000) was plotted.

Secondly, probability sensitivity analyses (PrSA) using 10,000 iterations to evaluate combined impact of all parameters uncertainty on model outcomes was performed. PrSA outcomes were plotted on the cost-effectiveness plane, used to calculate the 95% credibility interval (€ NMB). For PrSA, the SE and distributions are shown in Tables 1 and 2 and Additional file 1: Tables S1 and S2.

Thirdly, threshold analysis was performed to evaluate threshold values of parameters until a certain model outcome was reached. This is performed by varying the values of the number of pN1_lim_ patients who were falsely diagnosed as iN1_ext_ by [^68^Ga]Ga-PSMA-11 PET/CT (FP) and the disutility of ePLND until a QALY gain was observed.

## Results

### Main outcome

Treatment planning based on [^68^Ga]Ga-PSMA-11 PET/CT (no ePLND in case of positive iN1 and iM1 [^68^Ga]Ga-PSMA-11 PET/CT and only ePLND in case of negative [^68^Ga]Ga-PSMA-11 PET/CT) resulted in cost-savings and an almost equal QoL, €674 saved and 0.011 QALY loss per patient (Table 3). The positive effect of [^68^Ga]Ga-PSMA-11 PET/CT was caused by abandoning both an ePLND and unnecessary treatment in iM1 patients, saving costs and resulting in higher QoL. The negative effect was caused by lower QoL and high costs in the false palliative state, due to pN1_lim_ patients being falsely diagnosed as iN1_ext_. These patients received subsequently palliative treatment instead of potentially curative therapy (undertreatment). Currently regarding QoL, the negative effects outweigh the positive effects. Putting results into perspective, an ICER of €58,825 and NMB of -€243 and QoL loss indicates that currently the treatment is not cost-effective. On average, patients would live for 14.25 years, together with €35,695 reduced cost and 10.271 QALY in standard of care.

**Table 3:** Deterministic, sensitivity and threshold results of the model for [^68^Ga]Ga-PSMA-11 PET/CT versus standard of care

	Incremental cost (€)	Incremental quality of life (QALY)	ICER (€/QALY)	Life years (years)	Net Monetary Benefit*	Incremental treatment cost (€) **	Incremental treatment quality of life (QALY) **
Standard of care (ePLND)***	€ 35,659	10.271		15.25		€15,586	− 0.07
*Strategy*							
PSMA PET/CT ([^68^Ga]Ga-PSMA-11 PET/CT)	− € 674	− 0.011	€58,825	- 0.02	− € 243	− € 757	+ 0.006
*Threshold analysis*							
N1_ext_ by [^68^Ga]Ga-PSMA-11 PET/CT (FP) = 0.8%	− € 631	0.0003	Dominant	− 0.005	€ 654	− € 656	+ 0.005
ePLND disutility = 0.052	− € 674	0.0002	Dominant	− 0.018	€ 694	− € 757	+ 0.018

*Net monetary benefit was calculated using a willingness to pay of €80,000 per QALY, for both increase and decrease of quality of life. *ePLND* extended pelvic lymph node dissection, *FP* false positive, *ICER* Incremental cost-effectiveness ratio, *IKNL*  the Netherlands Comprehensive Cancer Organisation, *PET/CT*  positron emission tomography/computed tomography, *PSMA * prostate specific membrane antigen, *QALY* quality-adjusted life years.

**Results from the decision table for treatment costs and effects

***For standard of care, the absolute costs and effects are shown

#### Deterministic sensitivity analysis

Additional file 1: Fig. S1 shows the impact of used input parameters on NMB. The results show that the parameter indicating false positive findings (pN1_lim_ patients who are falsely being diagnosed as iN1_ext_ patients by [^68^Ga]Ga-PSMA-11 PET/CT) is the most important parameter used in the model. A decrease in this parameter increases cost-effectiveness of [^68^Ga]Ga-PSMA-11 PET/CT implementation in our cohort.

#### Probability sensitivity analysis

**Fig. 3:**
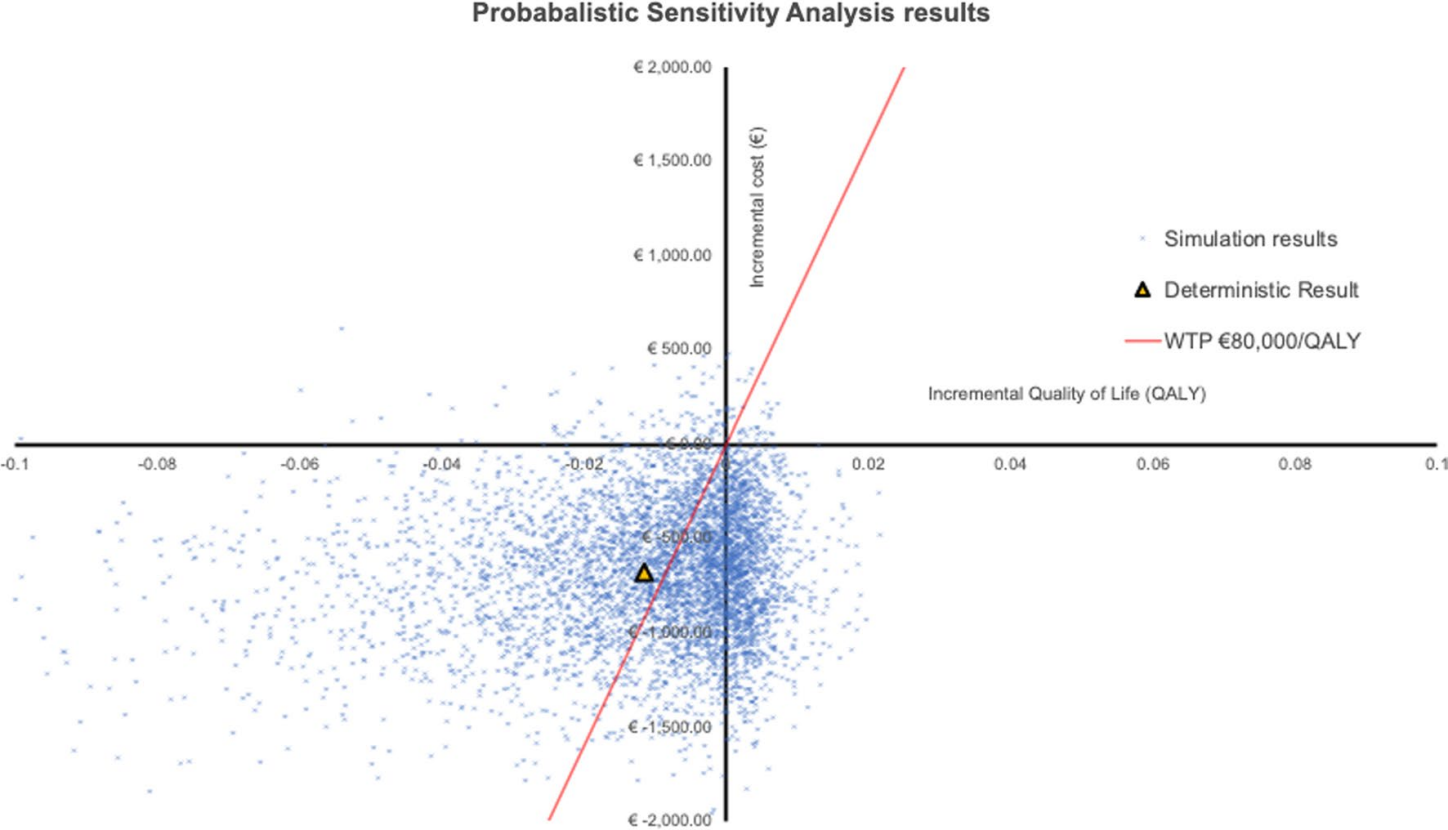
PrSA bootstrap analysis of 10,000 samples on cost-effectiveness of [^68^Ga]Ga-PSMA-11 PET/CT versus standard of care, plotted on the cost-effectiveness plane (incremental utility versus incremental cost). The triangle reflects the deterministic result. Results are mainly in the south-west quadrant, indicating a reduction in quality of life and cost-savings. *PET/CT* Positron emission tomography/computed tomography, *PrSA* probabilistic sensitivity analysis *PSMA* prostate specific membrane antigen, *QALY* quality-adjusted life years

Results of the PrSA are shown in Fig. 3. Sensitivity analysis showed inconclusiveness in cost-effectiveness for [^68^Ga]Ga-PSMA-11 PET/CT replacing ePLND in iN1 patients, with a 95% credibility interval for NMB between -€4,048 and €1,568 per patient.

#### Threshold analysis

Currently, this PSMA PET/CT strategy results in cost-savings and small QoL losses. Threshold analysis was performed to investigate when the strategy would result in QoL gain. Firstly, when the proportion of pN1_lim_ patients who were falsely diagnosed as iN1_ext_ by [^68^Ga]Ga-PSMA-11 PET/CT (FP) is reduced to <  ± 0.8%, the strategy results in QoL gain. Furthermore, when ePLND disutility is 0.052 QALY or higher, the strategy also results in QoL gain (Table 3, Additional file 1: Fig. S3). This concludes that improving the [^68^Ga]Ga-PSMA-11 PET/CT diagnostic sensitivity or more data on the disadvantages of the ePLND could reveal a cost-effective strategy.

## Discussion

This study evaluated the cost-effectiveness of treatment planning based on [^68^Ga]Ga-PSMA-11 PET/CT for primary staging in patients with prostate cancer.

Firstly, treatment planning based on [^68^Ga]Ga-PSMA-11 PET/CT instead of standard ePLND is cost-saving (€674) and results in minimal QoL loss (-0.011 QALY). The cost-saving is mostly due to improved iM1 detection of the [^68^Ga]Ga-PSMA-11 PET/CT compared to conventional imaging. The QoL loss is mostly as a result of the unwanted effects of extra investment costs in the false palliative state (pN1_lim_ patients being falsely diagnosed as iN1_ext_ leading to undertreatment).

Secondly, when the probability of false positive findings (resulting in palliative care) is reduced by <  ± 0.8% or when the disutility of ePLND proves to be > 0.052, [^68^Ga]Ga-PSMA-11 PET/CT is expected to increase QoL, while still saving costs. This indicates the high potential for cost-effectiveness of this technique. Extended PLND has been described to cause a 10-years QALY loss of ~ 0.07 [9]. Thus, eliminating unnecessary ePLND in iN1 or iM1 patients with PSMA PET/CT (as proposed in our model) has potential for health care costs savings in the general population.

Nevertheless, it is under debate whether interventions that reduce both costs and QoL can be cost-effective and if the same ICER values are applicable for this situation [14]. Therefore, we conclude the chosen strategy that is currently indecisive regarding cost-effectiveness. When a gain in QoL is achieved, the strategy is regarded cost-effective.

Regarding QoL, we need to consider the false positive findings (pN1_lim_ patients being falsely diagnosed as iN1_ext_) on the [^68^Ga]Ga-PSMA-11 PET/CT. This can potentially lead to undertreatment, meaning that a patient is not treated with curative intent but palliative. However, in current practice there is no strict delineation in the treatment choice. Choices are often made with shared decision making, and well-informed men with iNl_ext_ can undergo a potentially curative therapy. Therefore, we expect that in current practice the actual number of undertreated patients will be less.

Scholte et al. evaluated the cost-effectiveness of PSMA PET/CT in primary staging of prostate cancer versus ePLND [10]. They found PSMA PET/CT to be cost-saving with € -3074 (95% CI €-3515-€-2330), but at the expense of a QALY loss of 0.07 (95% CI -0.13-0.02), when ePLND was considered the gold standard with a sensitivity and specificity of 100%. Additionally, they showed that PSMA PET/CT would become cost-effective if an ePLND would account for a QoL loss of > 0.06. Our results are in line with these findings, indicating cost-savings and a small loss in QoL. Furthermore, our results indicate that [^68^Ga]Ga-PSMA-11 PET/CT becomes the dominant strategy when the ePLND has a QoL loss of > 0.052. However, Scholte et al. evaluated the total replacement of ePLND with PSMA PET/CT and did not include the ability of PSMA PET/CT to detect distant metastases. They also assumed that ePLND did not impact QoL and the diagnostic accuracy of PSMA PET/CT was based on literature only. Our study provides a more complete and realistic evaluation of the [^68^Ga]Ga-PSMA-11 PET/CT in clinical practice by not completely replacing ePLND by a PSMA PET/CT for lymph node diagnostics, but integrating ePLND as an adjunct to PSMA PET/CT due to the low sensitivity of PSMA PET/CT (as shown in previous prospective studies) [5–7].

Our model design has some limitations. First, palliative state was relatively heterogeneous, with patients staying in this state until death (mostly from natural causes, only ± 3% yearly mortality due to prostate cancer). However, DSA analysis showed little impact of utility and cost values of the palliative state. Second, ePLND was assumed the gold standard for lymph node metastases diagnosis. However, the performance of an ePNLD was likely overestimated. For example, in some cases [^68^Ga]Ga-PSMA-11 PET/CT assisted in extending the ePLND template, improving the diagnostic accuracy of an ePLND. Thus, in our model, ePLND diagnosis was assumed to be correct for all patients, except for M1 patients. Third, our model assumed that a false palliative state would fully resemble the costs of a true palliative state. It is likely that palliative care could be more beneficial in false palliative patients, as disease progression is overestimated here. Thus, real cost-effectiveness of the cohort was conservatively estimated and could be slightly higher than modelled in this study (Additional file 1: Fig. S3). Fourth, our model assumed that diagnosis of patients suffering from pM1a/b/c disease by [^68^Ga]Ga-PSMA-11 PET/CT was always correct. This assumption was due to additional diagnostics being required to confirm M1 findings and thereby exclude false positive findings. No extra costs were modelled for additional diagnostic investigations.

Fifth, the calculations of cost-effectiveness in this study are based on the Dutch health care.

system. However, in the online accessible tool mentioned in the method section you can adjust the cost and (dis)utility to compute your own cost-effectiveness results.

Finally, this analysis was based on a prospective cohort that excluded all patients with bone metastases on prior skeletal scintigraphy. Patients with a positive skeletal scintigraphy were not accounted for in our model. However, we estimated that [^68^Ga]Ga-PSMA-11 PET/CT would still be cost-effective, based on low prevalence of bone metastases at initial staging of intermediate- to high-risk prostate cancer patients [15]. Furthermore, this study only included patients with a Memorial Sloan Kettering Cancer Center (MSKCC)-risk > 10%. It can be expected that a lower threshold would result in a less cost-effective strategy. Patients with a lower MSKCC-score are more likely to have N0 disease and would still receive an ePLND in the proposed PSMA PET/CT strategy.

This study evaluated cost-effectiveness of a hypothetic implementation of [^68^Ga]Ga-PSMA-11 PET/CT as a substitute for ePLND in case of N1 and/or M1 disease on PET/CT. However, this is just one of the potential strategies of the implementation of [^68^Ga]Ga-PSMA-11 PET/CT in primary prostate cancer diagnostics and treatment planning. One may also choose to only perform an ePLND if PSMA PET/CT is positive for pelvic lymph node metastasis with the aim for a potential therapeutic effect, yet this remains debatable [1, 4]. In case of a negative pelvic PSMA PET/CT, an ePLND could be withheld knowing that a false negative PSMA PET/CT mostly concerns small lymph node metastasis [7]. The clinical outcome of this strategy also remains unknown [16]. With development of a dynamic PSMA PET/CT, more information can be obtained to increase scan accuracy for (distant) metastases detection [17]. Also this study only evaluated the [^68^Ga]Ga-PSMA-11 tracer although more tracers are available for the PSMA PET/CT with different accuracies and costs [18]. Next to ^68^ Ga-labelled PSMA, the most commonly used tracer is F-labelled PSMA (i.e. ^18^F-DCFPyL and ^18^F-PSMA-1007) with a sensitivity and specificity of about 41.2–73.5% and 94.0–99.4% [19, 20]. Although the accuracies are relatively close to each other we expect that the main difference in costs is due to difference in the production and transfer process [18].

## Conclusion

Initial prostate cancer staging and treatment planning based on [^68^Ga]Ga-PSMA-11 PET/CT instead of conventional diagnostics, in which ePLND is only performed in case of PSMA positive pelvic nodes, saves cost but results in small QALY loss due to the rate of false positive findings.

## Supplementary Information

**Additional file 1.** Additional figures; Additional tables.

## Data Availability

The datasets used and/or analysed during the current study are available from the corresponding author on reasonable request.

## Abbreviations

ADT: Androgen deprivation therapy
BCR: Biochemical recurrence
CBS: Centraal bureau voor statistiek (Dutch national price index)
CT: Computed tomography
DSA: Deterministic sensitivity analysis
ePLND: Extended pelvic lymph node dissection
FP: False positive
ICER: Incremental cost-effectiveness ratios
IKNL: The Netherlands Comprehensive Cancer Organisation
LNM: Lymph node metastases
M1: Distant metastasis
M1a: Extra pelvic lymph node metastasis
M1b/c: Bone and/or visceral metastasis
MRI: Magnetic resonance imaging
MSKCC: Memorial Sloan Kettering Cancer Center
N0: No lymph node metastasis
N1ext: Extended lymph nodes metastasis defined as > 4 pelvic lymph node metastases
N1lim: Limited lymph nodes metastasis defined as ≤ 4 pelvic lymph node metastases
NEOD: No evidence of disease
NMB: Net monetary benefit
p: Probability
PET: Positron emission tomography
PrSA: Probability sensitivity analyses
PSMA: Prostate specific membrane antigen
QoL: Quality of life
RP: Radical prostatectomy
RT: Radiotherapy
SE: Standard error
WTP: Willingness to pay
