# Supplementary material for: Cost-effectiveness of the implementation of [68Ga]Ga-PSMA-11 PET/CT at initial prostate cancer staging
Source: Insights Imaging. 2022 Aug 13;13:132. doi: 10.1186/s13244-022-01265-w (PMC9375809; doi:10.1186/s13244-022-01265-w)
Supplement: Supplementary file 1 — Additional file 1. Additional figures; Additional tables. [file 13244_2022_1265_MOESM1_ESM.docx]

**ELECTRONIC SUPPLEMENTARY MATERIAL**

**Cost-effectiveness of the implementation of [68Ga]Ga-PSMA PET/CT at initial prostate cancer staging**

**Additional figures**


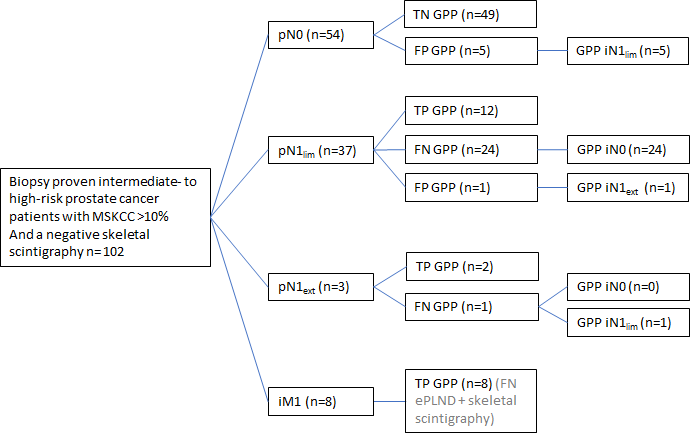


Figure S1 Patients outcomes from the PEPPER-study. Most of the iM1 patients had additional diagnostics (e.a. biopsy, additional imaging) to confirm M1 lesion. No lymph node metastasis (N0), limited lymph nodes metastasis defined as less than or equal to four pelvic lymph node metastasis (N1_lim_), extended lymph nodes metastasis defined as more than four pelvic lymph node metastasis (N1_ext_), and distant metastasis including extra pelvic lymph node metastasis, bone and/or visceral metastasis (M1).

Legend: FN = false negative, FP = false positive, GPP = [^68^Ga]Ga-PSMA-11 PET/CT, MSKCC = Memorial Sloan Kettering Cancer Center, PSA = Prostate specific antigen, PET/CT = Positron emission tomography/computed tomography, PSMA = Prostate specific membrane antigen, TN = true negative, TP= true positive.

Figure S2 The deterministic sensitivity results from the cost-effectiveness model versus the net monetary benefit. The parameters are sorted descending based on their impact on cost effectiveness outcomes. The x-axis indicates the effect the positive and negative estimate (stated on the end of the bar) of the parameter value has on cost effectiveness (NMB). The total length of both bars show the total potential impact on results. This figure indicates that GT: N1Lim – [^68^Ga]Ga-PSMA PET/CT: N1_Ext_ (FP) clearly the most important parameter of the analysis.

Legend: ADT = Androgen deprivation therapy, BCR = biochemical recurrence, ePLND = extended pelvic lymph node dissection, FN = False negative, FP = False positive, MRI = Magnetic resonance imaging, M1 = Distant metastasis including extra pelvic lymph node metastasis, bone and/or visceral metastasis, NOED = No evidence of disease, N0= No lymph node metastasis, N1_lim_ = limited lymph nodes metastasis defined as less than or equal to four pelvic lymph node metastasis, N1_ext_ = extended lymph nodes metastasis defined as more than four pelvic lymph node metastasis, P = Proportion, PET/CT = Positron emission tomography/computed tomography, PSMA = Prostate specific membrane antigen, QALY = Quality adjusted life years.

Figure S3 Threshold analysis for the parameters disutility ePLND and the proportion of N1_lim_ patients being falsely diagnosed as N1_ext_ by [^68^Ga]Ga-PSMA-11 PET/CT. Legend: ePLND = extended pelvic lymph node dissection, GPP = [^68^Ga]Ga-PSMA-11 PET/CT, GT= Ground true, N1_lim_ = limited lymph nodes metastasis defined as less than or equal to four pelvic lymph node metastasis, N1_ext_ = extended lymph nodes metastasis defined as more than four pelvic lymph node metastasis, PET/CT = Positron emission tomography/computed tomography, PSMA = Prostate specific membrane antigen, QALY = Quality adjusted life years, WTP = willingness to pay.

**Additional tables**

Table S1 Costs and disutilities

|  | | Value | Distribution (SE) | Source |
| --- | --- | --- | --- | --- |
| Treatment costs | | | | |
|  | Cost MRI/CT | €333 | Gamma (€33) | NZA Tariffs [1] |
|  | Cost [^68^Ga]Ga-PSMA-11 PET/CT | €1528 | Gamma (€153) | Internal Registry |
|  | Cost Skeletal scintigraphy | €664 | Gamma (€66) | Internal Registry |
|  | Cost ePLND | €7,360 | Gamma (€736) | OpenDisData [2] |
|  | Cost RP (without ePLND) | €12,005 | Gamma (€1,201) | OpenDisData [2] |
|  | Cost RP (parallel to ePLND) | €6,360 | Gamma (€636) | OpenDisData [2] |
|  | Cost RT | €3,346 | Gamma (€335) | Schwenk et al. [3] |
|  | Cost Pelvic RT | €2,818 | Gamma (€282) | Schwenk et al. [3] |
|  | Cost 3 years ADT | €3,801 | Gamma (€380) | FK [4] |
| Treatment disutilities (first year) | | | | |
|  | Disutility ePLND | 0.0108 | Beta (0.0108) | Appendix B |
|  | Disutility RP | 0.059* | Beta (0.0059) | Heijnsdijk et al. [5] |
|  | Disutility RT | 0.04* | Beta (0.004) | Heijnsijk et al. [5] |
|  | Disutility ADT | 0.022 | Beta (0.0022) | Sathianathen et al. [6] |
| Proportions | | | | |
|  | Proportion RP | 0.46 | Beta (0.046) | Scholte et al. [7] |
|  | Proportion RT | 1 – Proportion Radical Prostatectomy |  |  |

All cost values were converted into 2020 prices using the Dutch national price index [8].
*Disutility was estimated by taking the difference between the reported utility for the treatment and the NEOD utility value. Legend: ADT = Androgen deprivation therapy, ePLND = extended pelvic lymph node dissection, MRI = Magnetic resonance imaging, NOED = No evidence of disease, PET/CT = Positron emission tomography/computed tomography, PSMA = Prostate specific membrane antigen, RP = Radical prostatectomy, RT = Radiotherapy.

Table S2 Estimates for disutility ePLND within the first year

|  | Proportion | Utility | Disutility (versus 0.852 - 70 year healthy) | Weighted impact | Source |
| --- | --- | --- | --- | --- | --- |
| Lymphocele | 0.066 | 0.73 | 0.122 | 0.0081 | Cheville et al. [9]  Loeb et al. [10] |
| DVT | 0.025 | 0.84 | 0.012 | 0.0003 | Briganti et al. [11]  Heidenreich et al. [12]  Locadia et al. [13] |
| PE | 0.011 | 0.63 | 0.222 | 0.0025 | Briganti et al. [11]  Heidenreigh et al. [12]  Locadia et al. [13] |
|  |  |  | Total estimated disutility | 0.0108 |  |

Legend: DVT = deep veneus tromosis, ePLND = extended pelvic lymph node dissection, PE = pulmonal embolism.

**References additional tables**

1. Nederlandse Zorgautoriteit. Tarieventabel dbc-zorgproducten en overige-zorgproducten per 1 januari 2019 [Internet]. 2019 [cited 02 July 2021]. Available from: <https://puc.overheid.nl/nza/doc/PUC_236092_22/1/>.

2. Nederlandse Zorgautoriteit. Open data van de Nederlandse Zorgautoriteit [Internet]. 2021 [cited 02 July 2021]. Available from: <https://www.opendisdata.nl/>.

3. Schwenck J, Olthof SC, Pfannenberg C, et al. (2019) Intention-to-Treat Analysis of (68)Ga-PSMA and (11)C-Choline PET/CT Versus CT for Prostate Cancer Recurrence After Surgery. J Nucl Med 60:1359-1365.

4. Farmacotherapeutisch kompas. Gonadoreline-agonisten [Internet]. [cited 02 July 2021]. Available from: <https://www.farmacotherapeutischkompas.nl/bladeren/groepsteksten/gonadoreline_agonisten>.

5. Heijnsdijk EA, Wever EM, Auvinen A, et al. (2012) Quality-of-life effects of prostate-specific antigen screening. N Engl J Med 367:595-605.

6. Sathianathen NJ, Alarid-Escudero F, Kuntz KM, et al. (2019) A Cost-effectiveness Analysis of Systemic Therapy for Metastatic Hormone-sensitive Prostate Cancer. Eur Urol Oncol 2:649-655.

7. Scholte M, Barentsz JO, Sedelaar JPM, Gotthardt M, Grutters JPC, Rovers MM (2020) Modelling Study with an Interactive Model Assessing the Cost-effectiveness of (68)Ga Prostate-specific Membrane Antigen Positron Emission Tomography/Computed Tomography and Nano Magnetic Resonance Imaging for the Detection of Pelvic Lymph Node Metastases in Patients with Primary Prostate Cancer. Eur Urol Focus 6:967-974.

8. Centraal bureau voor statistiek. Consumentenprijzen; prijsindex 2015=100 [Internet]. 2021 [cited 02 July 2021]. Available from: <https://opendata.cbs.nl/statline/?dl=3F0E#/CBS/nl/dataset/83131NED/table>.

9. Cheville AL, Almoza M, Courmier JN, Basford JR (2010) A prospective cohort study defining utilities using time trade-offs and the Euroqol-5D to assess the impact of cancer-related lymphedema. Cancer 116:3722-3731.

10. Loeb S, Partin AW, Schaeffer EM (2010) Complications of pelvic lymphadenectomy: do the risks outweigh the benefits? Rev Urol 12:20-24.

11. Briganti A, Chun FK, Salonia A, et al. (2006) Complications and other surgical outcomes associated with extended pelvic lymphadenectomy in men with localized prostate cancer. Eur Urol 50:1006-1013.

12. Heidenreich A, Varga Z, Von Knobloch R (2002) Extended pelvic lymphadenectomy in patients undergoing radical prostatectomy: high incidence of lymph node metastasis. J Urol 167:1681-1686.

13. Locadia M, Bossuyt PM, Stalmeier PF, et al. (2004) Treatment of venous thromboembolism with vitamin K antagonists: patients' health state valuations and treatment preferences. Thromb Haemost 92:1336-1341.

**Cost-effectiveness Dutch population**

To assess population wide cost-effectiveness of the [^68^Ga]Ga-PSMA-11 PET/CT in the Dutch population, data from the Netherlands Comprehensive Cancer Organisation (IKNL) [1] were supplied (Dutch registered prostate cancer population (intermediate- to high-risk prostate cancer patients with histopathologic confirmed metastasis in the year 2019 in the Netherlands)). We assumed that the percentage of additional distant metastases by the [^68^Ga]Ga-PSMA-11 PET/CT in the PEPPER-study cohort would remain equal resulting in the following population wide percentages: 82.6% pN0, 9.0% pN1_lim_, 0.6% pN1_ext_, and 7.8% iM1.

In the Dutch population, the PSMA PET/CT strategy results in cost savings (€89) and QoL gain (0.00003 QALY). This results in a positive cost-effective strategy given a dominant ICER and a positive NMB of €91 (table S1).

|  | Incremental cost (€) | Incremental quality of life (QALY) | ICER (€/QALY) | Life years  (years) | | Net Monetary Benefit* | Incremental  treatment cost (€) ** | Incremental treatment quality of life (QALY) ** |
| --- | --- | --- | --- | --- | --- | --- | --- | --- |
| Standard of care (ePLND)*** | € 22,497 | 11.09 |  | | 16.01 |  | €13,897 | - 0.06 |
| Strategy: | | | | | | | | |
| Population wide (IKNL-population) | - € 89 | 0.00003 | Dominant | | - 0.004 | € 91 | - € 109 | 0.004 |

Table S1 Cost-effectiveness results Dutch population

*Net monetary benefit was calculated using a willingness to pay of €80,000 per QALY, for both increase and decrease of quality of life.

**Results from the decision table for treatment costs and effects.

*** For standard of care the absolute costs and effects are shown.

Legend: ICER = Incremental cost-effectiveness ratio, IKNL = the Netherlands Comprehensive Cancer Organisation, QALY = Quality adjusted life years.

**References Cost-effectiveness Dutch population**

1. Centraal bureau voor statistiek. Levensverwachting; geslacht, leeftijd (per jaar en periode van vijf jaren) [Internet]. 2021 [cited 02 July 2021]. Available from: <https://opendata.cbs.nl/statline/#/CBS/nl/dataset/37360ned/table?fromstatweb>.
